# Modulation of neurofluid fluctuation frequency by baseline carbon dioxide in awake humans: the role of the autonomic nervous system

**DOI:** 10.3389/fphys.2026.1750101

**Published:** 2026-02-18

**Authors:** Xiaole Z. Zhong, Catie Chang, J. Jean Chen

**Affiliations:** 1 Rotman Research Institute at Baycrest, Toronto, ON, Canada; 2 Department of Medical Biophysics, University of Toronto, Toronto, ON, Canada; 3 Department of Electrical and Computer Engineering, Vanderbilt University, Nashville, TN, United States; 4 Department of Computer Science, Vanderbilt University, Nashville, TN, United States; 5 Department of Biomedical Engineering, Vanderbilt University, Nashville, TN, United States; 6 Department of Biomedical Engineering, University of Toronto, Toronto, ON, Canada

**Keywords:** autonomic nervous system, carbon dioxide, cerebrospinal fluid, frequency modulation, heart rate variability, respiratory volume pertime, sympathetic activity

## Abstract

**Introduction:**

Cerebrospinal fluid (CSF) pulsations are linked to hemodynamics, with autonomic mechanisms, suggested to modulate slow-wave induced pulsations.

**Method:**

To explore autonomic regulation’s role in neurofluid flow, independent of sleep and neural activity, we hypothesized that modulating basal CO_2_ (altering vascular tone, cardiac activity and respiration) would highlight this link.

**Results:**

Using resting-state BOLD fMRI in neurofluid regions under different CO_2_ levels (capnic states), we found: 1) biomechanical modulation does not explain neurofluid dynamic variations across capnias; 2) beyond respiration, heart-rate variability independently drives low-frequency neurofluid flow, indicating autonomic control; 3) altered CO_2_ primarily affects neurofluid dynamics through the frequency (and not amplitude) of heart-rate and respiratory-volume variability.

**Discussion:**

These results suggest that both hyper- and hypocapnia disrupt how CSF responds to autonomic regulation, seen in deviations from normal cardiac and respiratory responses. Our work reveals neurofluid dynamics’ sensitivity to CO_2_’s frequency response, best explained by autonomic modulation. Modulating basal CO_2_ offers a new way to influence human neurofluid dynamics, independent of sleep or neuronal activity.

## Introduction

An understanding of neurofluid dynamics has been gaining importance, in part given the link between neurofluid dynamics and glymphatic flow (Taoka and Naganawa, 2020), which has in turn been linked to dementia pathology (Song et al., 2023). Neurofluids are defined as “fluids in which the central nervous system is immersed, such as blood, cerebrospinal fluid (CSF) and interstitial fluid (ISF)” (Taoka and Naganawa, 2020). Based on the current understanding, the driver of barogenic CSF flow is arterial pulsation. The drivers of this arterial pulsation predominantly originate from vasomotion, respiration and cardiac activity (Rasmussen et al., 2022). The amplitude of low-frequency CSF fluctuations, in particular, has been related to glymphatic flow, but the exact interaction between CSF fluctuations and glymphatic flow remains unclear. It has been suggested that, in addition to CSF fluctuation amplitude, CSF fluctuation frequency is linked with distinct mechanisms driving CSF fluctuation. The low-frequency CSF fluctuation that drives glymphatic flow is driven by vascular oscillators which can be further subdivided into endogenic (0.001–0.02 Hz), neurogenic (0.02–0.04 Hz) and vasogenic (0.06–0.14 Hz) oscillations (Bracic and Stefanovska, 1998). Myogenic oscillations are driven by Mayer waves, while neurogenic oscillations are primarily the result of innervation of the autonomic nervous system on the microvasculature (Strik et al., 2002).

Being sensitive to blood and CSF flow, blood-oxygenation-level-dependent (BOLD) resting-state fMRI (rs-fMRI) has recently enhanced the feasibility of broadening the study of neurofluid dynamics (Fultz et al., 2019; Yang et al., 2022; Diorio et al., 2023). Using BOLD, recent studies have highlighted the strong modulatory effects of sleep on CSF flow (Fultz et al., 2019). Specifically, Fultz et al. attributed CSF pulsations to widespread changes in hemodynamics (hemodynamic) driven by sleep-state slow-wave electrocortical activity in predominantly N1 and N2 sleep stages (Fultz et al., 2019). Indeed, hemodynamic and CSF oscillations, two main types of neurofluid dynamics, were recently found to be linked through a temporal derivative (Yang et al., 2022). Interestingly, Williams et al. later found CSF flow to be coupled to localized visual activity in the awake state, cementing the role of neural activity irrespective of sleep (Williams et al., 2023). However, widespread hemodynamic (and by inference CSF flow) modulations can be achieved in the awake state through myoactive mechanisms independently of electrocortical activity (Wang et al., 2022), including by modulating intravascular carbon dioxide (CO_2_) (Halani et al., 2015).

The primary biomechanical driver of hemodynamic fluctuations and hence CSF flow remains arterial pulsation (Rasmussen et al., 2022), it is reasonable to expect CSF fluctuation amplitude to be related to vascular tone. That is, the manner in which the rhythmic pressure waves generated by the heartbeat in the arteries are transmitted and modified in the CSF system depends on biomechanical factors such as brain-tissue compliance and vascular reactivity. Thus, at reduced vascular tone, the ability of hemodynamic to respond to arterial pulsation is reduced, resulting in reduced ability to induce CSF fluctuations. CO_2_ is a potent vasodilator and thereby modulates the effect of arterial pulsation. Therefore, CO_2_ modulations have been used to achieve vascular-tone modulation through the effect on vascular smooth muscles (Xie et al., 2006). Hypercapnia can achieve vasodilation (Kety and Schmidt, 1948; Reivich, 1964), and hypocapnia vasoconstriction. Both hyper- and hypocapnia result in diminished vascular tone (as measured by BOLD-fMRI) and hence reduced BOLD signal amplitude (Halani et al., 2015). This reduced vascular tone can also be reflected in changes in the frequency characteristics of the rs-fMRI response (Cohen et al., 2002). Moreover, hypercapnia is known to increase hemodynamic and decrease CSF volume (van der Kleij et al., 2020). Thus, there is ample evidence to support hypercapnia and hypocapnia modulating brain hemodynamics. This is analogous to the case of hypertension, whereby brain hemodynamics modulated by blood pressure (Sugimori et al., 1994). Thus, characterizing the effect of basal CO_2_ on the arterial-CSF system may help us understand biomechanical modulation of CSF flow.

As an alternative mechanism, recent work has proposed that the autonomic nervous system (ANS) plays an important role in CSF modulation (Picchioni et al., 2022). In particular, since respiration is controlled by the activity of the ANS (Tattersfield and McNicol, 1987), and since deep breaths reliably modulate global brain hemodynamics, Picchioni et al. used a cued deep-breathing task to transiently lower CO_2_ and produce an increase in CSF pulsations with lag times consistent with a mechanism involving the autonomic nervous system (ANS) (Picchioni et al., 2022). Moreover, respiratory modulation has been shown to produce CSF net displacement in the brain and spinal cord (Liu et al., 2025). As the ANS can directly control CBV, ANS activity can also alter vascular tone, and ANS tone is directly related to baseline CO_2_ (Fukuda et al., 1989; Polosa et al., 1983). Indeed, the critical role of the ANS in controlling vasoconstriction is well established (Zhang et al., 2002). Accordingly, the work of Picchioni et al. suggests that beyond the previously reported mechanism of neurofluid flow linked to CBV that is driven by electrocortical activity, CBV is also driven by ANS regulation through sympathetic control of respiration and vascular tone. Indeed, given the fact that changes in pulse volume, sympathetic activity, and inspiratory depth are known to co-occur with CSF fluctuations (especially in the neurogenic band) and with the K-complexes that are characteristic of N2 sleep (Colrain, 2005; Halász et al., 2004; de Zambotti et al., 2018), ANS regulation may also mediate the CSF pulsations during sleep. However, it is unclear whether the ANS can modulate CSF flow not only through respiration, but also through cardiac pulsation. This, in turn, may be modulated by the biomechanical properties of the blood vessels that vary with basal CO_2_.

Beyond vascular tone and respiratory modulation, ANS could potentially modulate CSF flow through other mechanisms. It was recently shown that the heart rate (HR) and respiratory rate (RR) are both closely related to CSF dynamics (Yang et al., 2024). High-frequency respiratory oscillation amplitude was found to correlate with phase of low-frequency CSF flow at 0.02 Hz (Vijayakrishnan Nair et al., 2022), which is the frequency peak associated with sympathetic control of blood flow (Kastrup et al., 1989). This finding is novel as it could broaden our understanding of neurofluid modulation from amplitude-based to phase/frequency based. Moreover, as mentioned earlier, hemodynamic fluctuations can be driven by electrocortical activity (Fultz et al., 2019; Yang et al., 2024), and CO_2_ can also modulate electrocortical excitability in addition to vascular tone and ANS tone (Driver et al., 2016; Xu et al., 2011). Thus, the effect of CO_2_ on neuronal activity fluctuations may modulate CSF fluctuations independently of the biomechanical and ANS factors.

**FIGURE 1:**
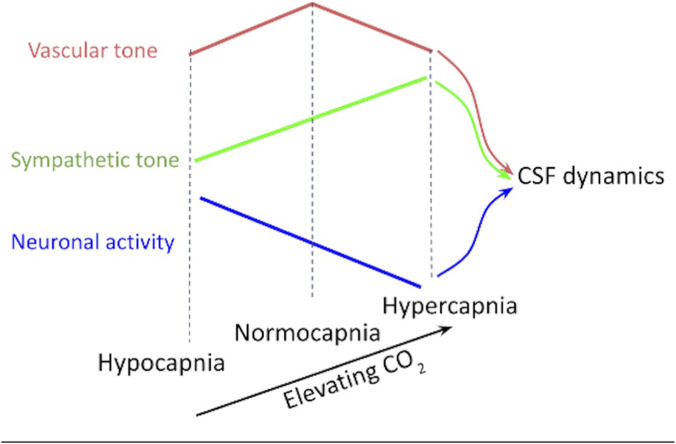
The predictions of CSF flow dynamics across capnias is based on three different physiological pathways: vascular tone, sympathetic tone, and neuronal activity. According to the vascular-tone theory, CSF fluctuations should be maximal at normocapnia. According to the neuronal-activity theory, CSF fluctuations should be maximized at hypocapnia. Lastly, according to the sympathetic-tone theory, CSF fluctuations should be maximized at hypercapnia. These theories will be tested using empirical data involving different capnias, at which all three variables will be altered.

Driven by the need to further clarify the contributions of different pathways to CSF fluctuations, this paper investigates which pattern (summarized in Figure 1) most strongly predicts CSF fluctuation dynamics across different CO_2_ levels. Our guiding questions are: 1) when baseline CO_2_ is manipulated, does the biomechanical, ANS or neuronal mechanism dominate the regulation of low-frequency neurofluid fluctuations? 2) Can the ANS modulate low-frequency neurofluid dynamics not only through respiration but also cardiac activity? 3) What roles does the frequency of CSF and vascular fluctuations play in the coordination between ANS-related activity and neurofluid dynamics? To address these questions, this work strives to manipulate biomechanics and ANS activity by manipulating baseline CO_2_ level in a group of healthy young adults. Specifically, hypercapnia increases sympathetic activity and hypocapnia increases parasympathetic activity. Therefore, manipulating baseline CO_2_ may result in the modulation of CSF dynamics via an ANS-mediated pathway in addition to the biomechanical- and neuronally mediated pathways. We characterize the contribution of ANS variables (related to respiration and cardiac pulsation) on CSF and vascular fluctuations as measured using BOLD.

## Methods

### Data set

We recruited 13 participants at hypercapnic, normocapnic and hypocapnic baselines, with further exclusion of two participants due to the absence of physiological measurements, resulting in 11 participants (25–38 years old; 2 males and 9 females) being included in the study. Participants were recruited through the Baycrest Participants Database, consisting of individuals from the Baycrest and local communities. The study was approved by the research ethics board (REB) of Baycrest, the experiments were performed with the understanding and written informed consent of each participant, according to REB guidelines.

### MRI acquisition

Image acquisition was performed with a Siemens TIM Trio 3 T System (Siemens, Forchheim, Germany), which employed 32-channel phased-array head coil reception and body-coil transmission. BOLD data was acquired using a gradient-echo EPI pulse sequence (TR = 380 ms, TE = 30 ms, FA = 40°, 15 slices, 3.44 × 3.44 × 5 mm^3^ with 20% slices gap, 950 volumes). T1-weighted MPRAGE anatomical image was acquired (TR = 2,400 ms, TE = 2.43 ms, FOV = 256 mm, TI = 1,000 ms, readout bandwidth = 180 Hz/px, voxel size = 1 × 1 × 1 mm^3^). A finger oximeter built into the scanner was used to monitor heart rate, and a pressure-sensitive belt was used to monitor respiration during the BOLD scan. Data sets without coverage of the aqueduct (n = 1) and fourth ventricle (n = 4) were excluded from subsequent analyses of aqueduct and fourth ventricle signals.

### Gas manipulation

We administered mixtures of O_2_, CO_2_ and medical air using the RespirAct^TM^ breathing circuit (Thornhill Research, Toronto, Canada) for all gas manipulations. With the sequential gas delivery method (Slessarev et al., 2007), the end-tidal partial pressure of O_2_ (PETO_2_) and CO_2_ (PETCO_2_) pressures were targeted by computerized and independent means. This setup allowed us to manipulate the basal PETCO_2_ level of each participant precisely, without altering PETO_2_. We separately targeted a normocapnic baseline (participant’s nature baseline), a hypercapnic baseline, and a hypocapnic baseline, separated by 4 mmHg CO_2_ (to avoid metabolic change (Chen and Pike, 2010)). There was no change in capnia during the recording in order to avoid the possibility of CSF directly being affected by vasodilation caused by the transition between different capnias (Liu et al., 2025; Zimmermann et al., 2023). The RR was self-regulated throughout the respiratory challenges. Hypocapnia is achieved with RespirAct^TM^ primarily through increased breathing depths, and hypercapnia is achieved by increasing CO_2_ levels in the air supply. A pseudo-randomized sequence of capnic conditions was used across different participants, with approximately two minutes between each condition. During the study, breath-by-breath CO_2_ levels were recorded at a rate of 50 Hz using the RespirAct.

### Data preprocessing and parameterization

The FreeSurfer reconstruction was performed on the T1 anatomical data for all participants using FreeSurfer 6.0 (available at: https://surfer.nmr.mgh.harvard.edu). This reconstruction provided tissue segmentation of gray matter, white matter structures, as well as ventricles, which can then be used to delineate regions of interest.

#### BOLD data processing

The BOLD data were preprocessed as follows: 1) discard the first 200 volumes, 2) motion coregistration, 3) motion regression with 6 parameters (3 translations and 3 rotations), 4) demeaning, 5) coregistration with the anatomical image.

#### Vascular signals

Vascular regions of interest (ROIs) were defined using the same method as our previous study (Attarpour et al., 2021). That is, arterial and venous maps were generated directly from BOLD data by delineating areas in the top 20 percentile of signal-fluctuation amplitudes. Arteries and veins were separated manually based on anatomical information. In large arteries, slow BOLD signal fluctuations are driven primarily by dynamic magnetic-susceptibility differences driven by dynamic partial-volume effects between the highly oxygenated arterial blood and surrounding tissue (Tong et al., 2019). In large veins, the rs-fMR signal fluctuations are largely attributable to transverse relaxation-rate variations, which, also as shown in our previous work (Attarpour et al., 2021), demonstrate patterns similar to those in large arteries.

#### CSF signals

**FIGURE 2:**
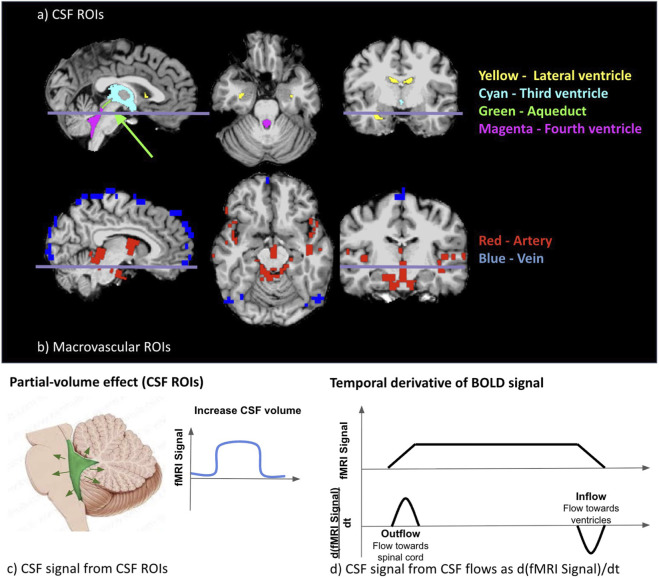
Illustration of the CSF ROI **(a)** and the vascular ROI **(b)** from a representative participant. A demonstration of the change in the fMRI signal associated with CSF ROIs **(c)** and flow **(d)**. ROIs in **(a,b)** were colored as described above, and the line indicates the position of the transverse slice. The CSF signal was obtained using two different approaches based on two different mechanisms. The fMRI signals increased in CSF ROIs corresponding to increases in the volume of CSF ROIs **(c)**. A positive BOLD-temporal-derivative CSF flow corresponds to the outflow of CSF (flow towards the spinal cord), while a negative temporal derivative of the fMRI signal corresponds to the inflow of CSF (flow towards the ventricles) **(d)**.

Following previous research, we also calculated a surrogate of CSF velocity variations by applying the first temporal derivative to the global-mean BOLD signal (GMS) that was detrended and filtered into 0.01–0.1 Hz frequency band; for this purpose, we used a zero-delay fourth-order Butterworth filter (Fultz et al., 2019; Yang et al., 2022). Moreover, BOLD signals in CSF-related ROIs were extracted. The CSF ROIs were derived either through the use of FreeSurfer tissue segmentation (for the lateral ventricle, third ventricle and fourth ventricle) or by manual delineation (for the cerebral aqueduct) and were downsampled to BOLD space for analysis. Fluctuation in BOLD signals from CSF ROIs reflected the variation in ventricular volume (Figure 2c, partial-voluming effects), whereas fluctuation of temporal derivative of global BOLD signal reflected the inflow and outflow of CSF flow (Figure 2d).

#### Autonomic signals

The explanatory variables related to the ANS include: (1) HR; (2) RR; (3) heart rate variability (HRV); (4) respiratory volume per time (RVT). These parameters are associated with ANS activity, as reported in previous studies (Gullett et al., 2023; Malik et al., 2019; Seals et al., 1990; Shams et al., 2022). Both HRV and RVT were estimated based on the method used in a previous study, with a window size of 4 s (Golestani et al., 2015). Moreover, recent work also found a link between global neuronal activity and the ANS, and accordingly, global-mean gray matter signal (GGMS) was also extracted as a surrogate of global neural activity (Bolt et al., 2022). Fluctuations in GGMS reflect changes in the cortical mean BOLD signal that are found to correlate with ANS variables such as pulse amplitude and respiratory volume (Tong et al., 2019).

#### Frequencies of interest

We identified frequency bands of interest that are consistent with those reported in previous work (Tong et al., 2019). The resulting bands were following and similar to previous reported ranges (Tong et al., 2019), suggesting analysis results can be extended to predefined frequency ranges:

1. Band 1: 0.01–0.14 Hz, represents neural activity and vasomotion;
2. Band 2: 0.14–0.56 Hz, reflects respiration;
3. Band 3: 0.56–1.31 Hz, reflects cardiac pulsation.

The actual range of each band was specific to the current group of participants and was determined in a data-driven approach with frequency-domain group-level independent component analysis (ICA) (Calhoun et al., 2001). Specifically, we extracted signals from the neurofluid ROIs of all participants and capnic conditions, and entered them into the ICA. Each band was defined by examining the power spectra of the first 20 independent components (ICs). The first three ICs that showed a similar spectral-peak frequency range to the given predefined frequency range were selected, based on which the boundaries of various frequency bands were determined, with no overlap between frequency bands. Interestingly, only three meaningful ICs (with noticeable distinctness from the rest of the ICs), corresponding to predefined frequency bands applied to all participants.

It was found that there is a modulation effect between respiratory measurements and low-frequency CSF flow (Vijayakrishnan Nair et al., 2022). Thus, we divided the BOLD signal in Band 1 into infraslow bands. Based on laser Doppler flowmetry, unstimulated blood flow in Band 1 exhibits 3 spectral peaks, namely:

1. The endogenic peaks (Band IS1): 0.001–0.02 Hz, represents an endogenic (metabolic) band related to the rhythmic regulation of vascular resistance to the blood flow triggered by variations in blood metabolic substrate concentration (Bracic and Stefanovska, 1998);
2. The neurogenic peak (Band IS2): 0.02–0.04 Hz, which may result from sympathetic neuronal activity (Kastrup et al., 1989);
3. The myogenic peak (Band IS3): 0.06–0.14 Hz, which is associated with blood-pressure regulation (Johnson, 1991), and is within the range of low-frequency HRV (Akselrod et al., 1981).

Following the formulation in (Zhong and Chen, 2022), we further calculated the normalized power (power normalized by signal temporal mean) and the central frequency of each frequency range, where the latter is computed as the frequency at the “centre of mass” of the spectrum, as follows:

$$centre\;frequency=\frac{\sum_{i=0}^{m}P_{i}f_{i}}{\sum_{i=0}^{m}P_{i}}$$ (1)

where *P* represents power, and *f* frequency, and (*i* = 0,…,*m*) is the frequency index in the Fourier domain (*m* corresponds to the index of the maximum frequency).

### Statistical analysis

#### Power spectra across capnic conditions

Power spectra were calculated using Welch’s method (Hamming window, 41 sample overlaps) and averaged across all participants for each capnic condition and each ROI.

#### Power and central frequency differences across capnic conditions

To demonstrate the difference between power and central frequency for three different capnic conditions, effect sizes were calculated for each band using Glass’s estimator (D) for each frequency band (Hedges, 1981; Cohen, 1977). The effects were further classified according to the standard set by the previous study (Sawilowsky, 2009) as follows: very small (D < 0.2), small (0.2 < D < 0.5), medium (0.5 < D < 0.8), large (0.8 < D < 1.2) and very large (D > 1.2).

#### Association between physiological factors and power and central frequency

An analysis of linear mixed effects was conducted to investigate the association between each physiological factor and the fMRI signal power and central frequency for each frequency band. The input parameters were all demeaned and normalized by their respective inter-participant standard deviation. The variable “ID” was included in the model as a random variable in order to account for repeated measures within each participant (identified by a participant ID number) (Equation 2 is in Table 1). Thus, the inter-participant variation is not of interest, as we are modeling the inter-capnic effects as the outcome measure. The model is illustrated in Table 1. Note that in Band 3, since HRV (Holland and Aboy, 2009) and RVT (Birn et al., 2006) are unlikely to extend into such a high-frequency band, only lower-frequency band metrics were incorporated into the linear mixed-effects model (*RVT*(*t*) power and frequency from Band 1 and *HRV*(*t*) power and frequency from Bands 1 and 2 were used as separate independent variables in the LMEs for all BOLD signal bands). All physiological parameters were included for each neurofluid signal metric. The 95% confidence interval of each coefficient in the model is determined by bootstrapping (10,000 iterations of resampling with replacement), and each coefficient is deemed significant only if its confidence interval does not span zero. Motion parameters (6 degrees of freedom) were also modeled using LME, confirming that no residual motion effects remained after motion regression.

**TABLE 1:** Details on the linear mixed effect models.

Y ∼ 1+(1|Participant ID)+∑X (Equation 2)			
Y (neurofluid signal)		X (physiological recordings)	
Power	Frequency	Power	Frequency
CSF flow	CSF flow	Respiratory rate (RR)	
Artery	Artery	Heart rate (HR)	
Vein	Vein	PETCO_2_	
Lateral ventricle	Lateral ventricle	Heart rate variability (P(HRV(t))	Heart rate variability (f(HRV(t))
Third ventricle	Third ventricle	Respiratory-volume per time (P(RVT(t))	Respiratory-volume per time (f(RVT(t))
Aqueduct	Aqueduct	• The 95% confidence interval with 10,000 bootstrapping • Significant only if its confidence interval does not span zero	
Fourth ventricle	Fourth ventricle		

Y represents the dependent variable and X the independent variable. A separate LME is constructed for each variable under the Y columns, using all variables under X columns (all x included for each Y, where power and frequency correspond to variables under the Y column).

#### Temporal characterization of ANS coordination: respiratory and cardiac response functions across capnias

The respiration response function (RRF) (Birn et al., 2008), which captures a change in fMRI signal in response to a change in RVT, was estimated to understand the mechanism by which the ANS affects fMRI signal dynamics through *RVT(t)*. For each participant, the mean signal from each neurofluid ROI as well as the GGMS and CSF velocity time series were deconvolved by the corresponding *RVT(t)* using the Laguerre expansion (Shams et al., 2022; Prokopiou et al., 2018) across three capnias. The cardiac response function (CRF) was also estimated to characterize the relationship between ANS-driven fMRI dynamics and *HRV(t)*. The paired two-tailed Wilcoxon signed rank test (p < 0.05; performed with MATLAB) was used to determine whether the peak height, lag, and full-width-half-maximum (FWHM) differed between pairs of capnias for the first and second peak as well as the centre frequency (Equation 1) for CRF and RRF.

#### Temporal characterization of vascular-tone response: arterial response function across capnias

To further separate the effects of ANS-induced effects on from biomechanical effects on CSF dynamics across capnias, the arterial transform function (ATF) was estimated using the same approach as CRF and RRF estimation, by substituting the *RVT(t)* and *HRV(t)* time courses with the arterial BOLD signal (See *Respiratory and cardiac response function across capnias* section). The paired two-tailed Wilcoxon signed rank test (p < 0.05; performed with MATLAB) was used to determine whether the peak height, lag, and FWHM differed between pairs of capnias for both the first peak as for *ATF(t)*.

#### Shared information between fMRI and respiratory/cardiac variability time series

To quantify the dynamic interaction between the fMRI and the ANS parameters *HRV(t)* and *RVT(t)*, we calculated the mutual information between these pairs of variables. This permitted us to assess the interactions unbiased by the accuracies of the response functions. Mutual information was calculated using a MATLAB toolbox (Feed et al., 2015) with the window width (k-nearest-neighbours) chosen to be consistent with the 0.1 Hz bandwidth of the BOLD signal (26 temporal samples). The mutual information index was normalized by the geometric mean. The paired two-tailed Wilcoxon signed rank test (p < 0.05; performed with MATLAB) was performed to determine whether there were differences between capnias.

## Results

**FIGURE 3:**
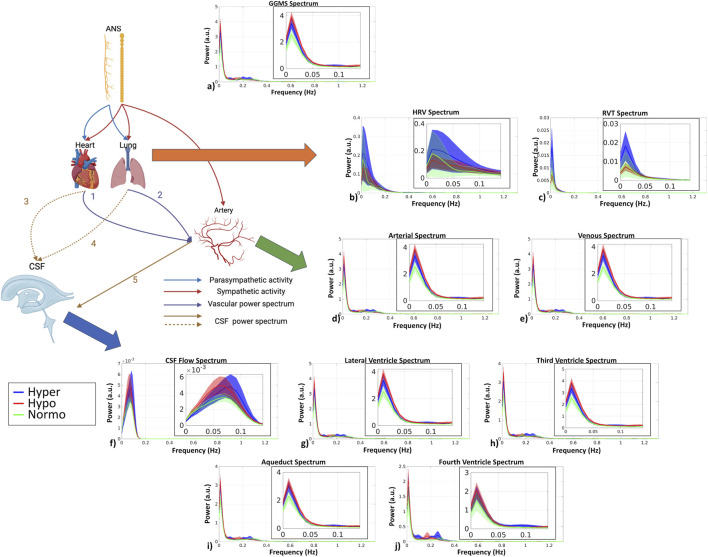
fMRI signal power spectra in all ROIs. The schematic illustrates the pathways under investigation that link the ANS to CSF dynamics. The heart and lung both receive parasympathetic (blue arrow) and sympathetic (red arrow) input, whereas the arterial vasculature only receives sympathetic input (red arrow). The lung and the heart may have a direct impact on arterial BOLD (purple arrows labeled “1” and “2”), which in turn directly drives CSF flow (brown arrow labeled “5”). CSF flow can also be driven indirectly by heart (blue dashed arrow labeled “3”) and lung (blue dashed arrow labeled “4”) activity. All power spectra were calculated after time-course normalization by the temporal mean. The shaded area represents standard error. The zoomed-in versions (Band 1) of the spectra are shown as insets. (Figure partially based on biorender: https://www.biorender.com/). **(a)** GGMS; **(b)** HRV; **(c)** RVT; **(d)** arterial ROI; **(e)** venous ROI; **(f)** CSF flow; **(g)** lateral ventricle; **(h)** third ventricle; **(i)** aqueduct; **(j)** fourth ventricle.

In Table 2, we illustrate the physiological metrics across the 2 capnic conditions, including PETCO_2_, heart rate (HR), and respiratory rate (RR). As expected, hypercapnia was associated with the highest PETCO_2_ levels, followed by normocapnia, with hypocapnia exhibiting the lowest levels. The highest HR was associated with hypercapnia, followed by hypocapnia, with the lowest HR associated with normocapnia. There was an opposite trend in the RR, where normocapnic RR was the highest, followed by hypercapnic and lastly hypocapnic RR. Significant differences across capnic levels were found for PETCO_2_ but not for the HR and RR in terms of group mean (not equivalent to pairwise differences). Additionally, Figure 3 shows power spectra for fMRI signals from vascular and CSF ROIs as well as HRV and RVT.

**TABLE 2:** Physiological measurements (mean and standard deviation).

Physiological measurements	Hypercapnia	Normocapnia	Hypocapnia	P-value
PETCO_2_ (mmHg)	42.91 ± 2.47	39.47 ± 1.93	36.44 ± 1.60	1.41e-07
Heart rate (Hz)	1.28 ± 0.32	1.15 ± 0.18	1.26 ± 0.31	0.54
Respiratory rate (Hz)	0.23 ± 0.07	0.23 ± 0.06	0.22 ± 0.06	0.93

P-values were computed by one-way analysis of variance (ANOVA).

### Vascular and CSF oscillations across different capnias

Signal metrics were compared across capnic conditions, and all associated results are shown as Cohen’s D values, as summarized quantitatively in Supplementary Figure S3 as well as verbally in Supplementary Table S1. When comparing hypocapnia to normocapnia, the fMRI signal in vascular and CSF ROIs behaved in very similar ways, and the same is true when comparing hypercapnia to normocapnia. Relative to normocapnia, both hypocapnia and hypercapnia are associated with increased fMRI signal power and shifts in BOLD signal frequency in a band-dependent manner. The behaviour of CSF velocity, only defined for frequencies up to 0.1 Hz, also mimics that of the fMRI signals in the vascular and CSF ROIs. Moreover, GGMS signal metrics behaved very similarly to those from vascular ROIs. For visualization purposes, the power spectra shown below was computed using Welch’s method (Hamming window, 41 sample overlaps), but the statistical results are based on the Fourier spectra.

### Drivers of vascular and CSF oscillations across capnic conditions

**FIGURE 4:**
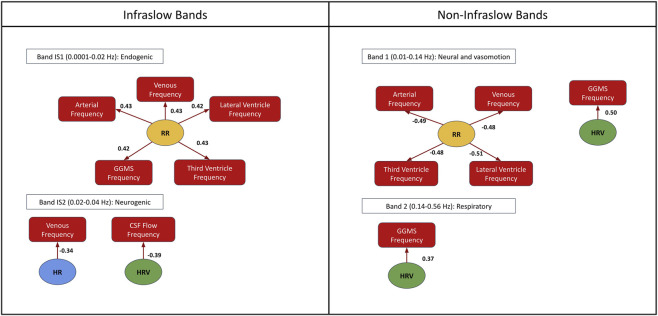
Factors linked to CSF signal fluctuations in the infraslow bands IS1-IS3 (left) and band 1–3 (right). Only significant results are shown (p < 0.05 with the bootstrapped test). The arrows indicate the significant association. The numbers displayed next to the arrows indicate the effect size. HR: heart rate; RR: respiratory rate; HRV: heart-rate variability.

We see obvious differences across the infraslow frequency bands and non-infraslow frequency bands (Figure 4). Interestingly, basal CO_2_ was not a strong driver of the differences across capnic conditions for all frequency bands. Below are the details of the effect for each band:

- Band IS1: Positive associations between frequency and RR in the artery, vein, lateral ventricle, third ventricle, and GGMS.
- Band IS2: Negative association between frequency and HR in veins; negative association between CSF flow frequency and HRV (f(*HRV*(*t*)).
- Band 1: Negative association between RR and frequency in arteries, veins, the lateral ventricle and third ventricle; positive association between frequency and HRV (f(*HRV*(*t*)) in the GGMS.
- Band 2: Positive association between HRV (f(*HRV*(*t*)) and GGMS frequency

It is also worth noting that extra-cranial sources in upper frequencies (Bands 2 and 3) do not significantly modulate neurofluid dynamics in our modeling framework. Moreover, we did not observe a direct modulatory effect of PETCO2 on neurofluid dynamics.

### RRF and CRF for different capnic conditions

**FIGURE 5:**
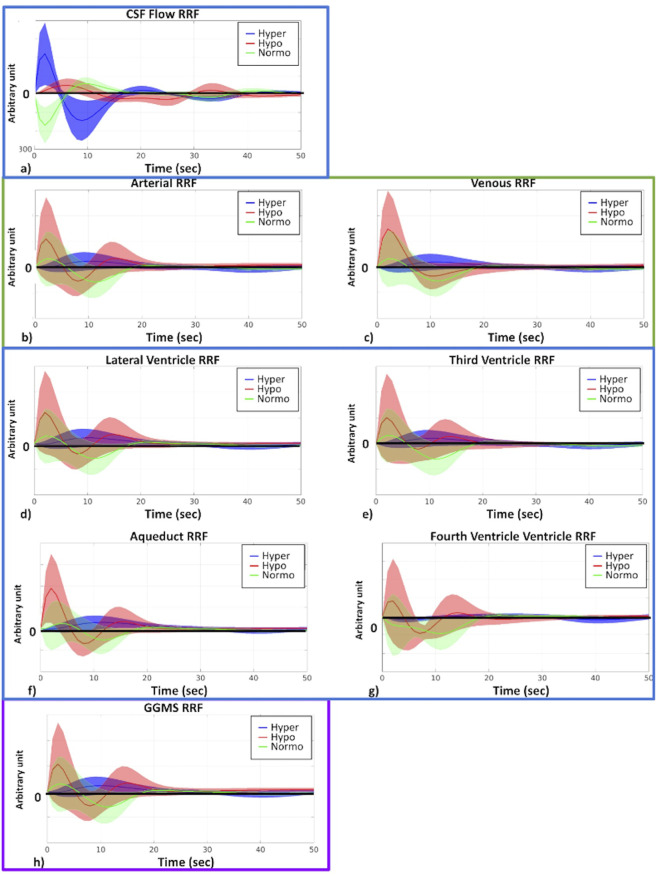
RRF across different capnias. All plots are shown as averages across participants, and the shaded area represents the standard error. The y-axis of plots is in arbitrary units. Hyper (blue): hypercapnia; hypo (red): hypocapnia; normo (green): normocapnia. **(a)** CSF flow; **(b)** arterial ROI; **(c)** venous ROI; **(d)** lateral ventricle; **(e)** third ventricle; **(f)** aqueduct; **(g)** fourth ventricle; **(h)** GGMS.

In this work, we use the RRF and CRF to characterize the time-dependent coordination between the ANS and CSF/vascular signals. Based on the fact that RR, HR and HRV are the most predominant mediators of the relationship between capnic condition and fMRI signal changes in neurofluid ROIs (as shown in Figure 4), we estimated the RRF linking respiratory volume per unit time to the fMRI data, as well as the CRF linking HRV to the fMRI data. As shown in Figure 5, there were distinguishable differences in the shapes of the RRF across three capnias. Further statistical analysis revealed that hypercapnia showed a higher first peak FWHM than normocapnia for all ROIs (excepting aqueduct; p_Artery_ = 0.03, p_vein_ = 0.02, p_LateralVentricle_ = 0.03, p_ThirdVentricle_ = 0.008, p_FourthVentricle_ = 0.03; D_Artery_ = 1.00, D_vein_ = 1.10, D_LateralVentricle_ = 1.00, D_ThirdVentricle_ = 1.10, D_FourthVentricle_ = 1.03) and GGMS (p_GGMS_ = 0.03; D_GGMS_ = 1.00), but not for CSF flow. The hypercapnia also showed higher first peak lags than hypocapnia for lateral and third ventricles (p_LateralVentricle_ = 0.04, p_ThirdVentricle_ = 0.02; D_LateralVentricle_ = 1.01, D_ThirdVentricle_ = 1.02).

**FIGURE 6:**
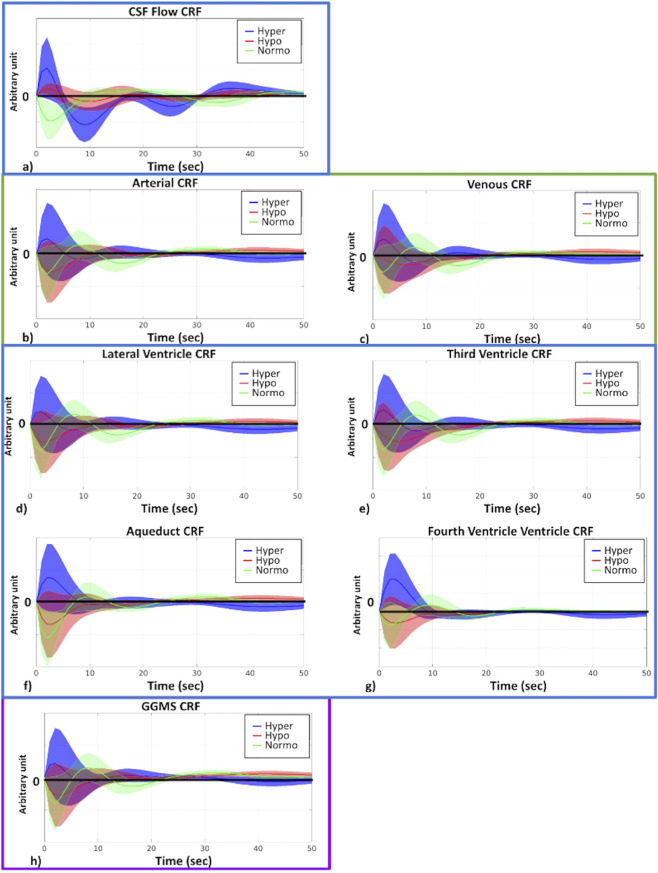
CRF across different capnias. All plots are shown as averages across participants, and the shaded area represents standard error. The y-axis of plots is in arbitrary units. Hyper (blue): hypercapnia; hypo (red): hypocapnia; normo (green): normocapnia. **(a)** CSF flow; **(b)** arterial ROI; **(c)** venous ROI; **(d)** lateral ventricle; **(e)** third ventricle; **(f)** aqueduct; **(g)** fourth ventricle; **(h)** GGMS.

As shown in Figure 6, there were distinguishable differences in the shapes of the CRF across three capnias. Further statistical analysis revealed that the first peak intensity for the CRF for hypocapnia was significantly higher than normocapnia for CSF flow (p_CSFFlow_ = 0.02; D_CSFFlow_ = 0.90). Moreover, normocapnia showed higher second peak intensity than hypocapnia for CSF flow (p_CSFFlow_ = 0.02; D_CSFFlow_ = 1.07).

### Arterial-transfer function (ATF) for different capnic conditions

**FIGURE 7:**
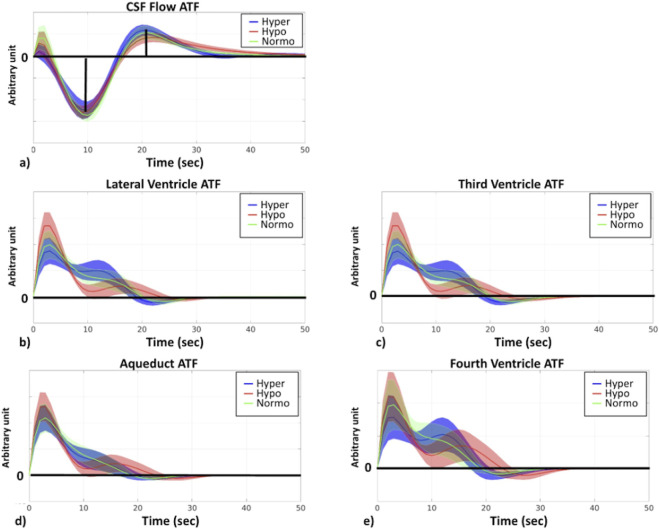
ATF across different capnias. All plots are shown as averages across participants, and the shaded area represents the standard error. The y-axis of plots is in arbitrary units. Hyper (blue): hypercapnia; hypo (red): hypocapnia; normo (green): normocapnia.

As the CSF flow dynamic in the resting state (after the capnic effect reaches a steady state) originates primarily from vascular activity, especially arteries, given their ability to dilate and contract, ATF was estimated in order to better understand how ANS-induced vascular tone differences impact CSF dynamics. The ATF quantitatively describes how arterial fluctuations are converted to CSF flow fluctuations, a process which is directly influenced by tissue elasticity and vascular tone, as described earlier. As outlined in the biophysical model (Zhong et al., 2024), the increase in arterial signal is mainly due to the contraction of the arteries (less R_2_’ decay caused by arterial-derived local-field inhomogeneity). As shown in Figure 7, ATFs from different CSF ROIs and CSF flow behave very similarly at different capnias. Further statistical analysis revealed there is no significant difference in the ATF parameters across different capnias, although qualitative differences exist in the plots for CSF ROIs.

**FIGURE 8:**
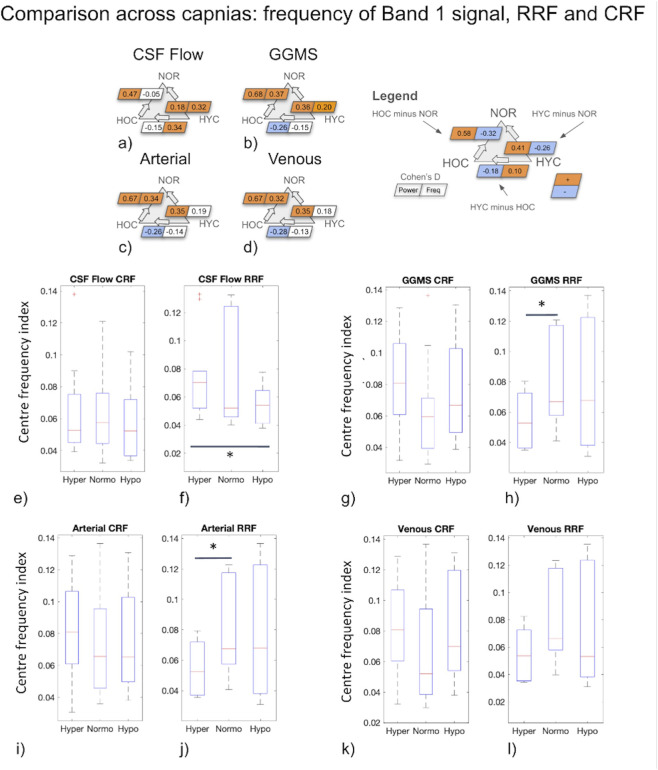
Summarized in **(a–d)** are the differences in power and centre-frequency index of Band 1 signal fluctuations in CSF flow, GGMS signal, the BOLD signal in the arterial and venous ROIs, respectively. Correspondingly, **(e–l)** summarize the centre-frequency indices of RRF and CRF calculated for CSF flow, the GGMS signal, the BOLD signals in the arterial and venous. Value presented in a-d represents Cohen’s D, thresholded with small effect (D > 0.2). Asterisks indicate significant differences based on the Wilcoxon signed test (p < 0.05; p_CSFFlow_ = 0.049, p_GGMS_ = 0.04, P_Artery_ = 0.04). HYC (Hyper): hypercapnia; HOC (Hypo): hypocapnia: NOR (Normo): normocapnia.

Given that the ATF did not differ significantly across capnias, whereas the RRF and CRF did, and given that we mainly uncovered differences in signal frequency across capnias (Figures 8a–d) that are mediated by *HRV*(*t*) and *RVT*(*t*) frequencies (Figure 4), we wanted to clarify if these frequency differences in Figures 8a–d are reflected in the RRF and CRF frequencies. As shown in Figures 8e–l, frequency differences across capnias were identified in the RRF estimates associated with all signals, but not in the CRF estimates. This suggests that despite *HRV*(*t*) frequency (and not *RVT*(*t*) frequency) being the major ANS-related mediator of neurofluid signal frequency differences across capnias, the frequency governing the propagation of HRV variations to neurofluids variations remains unaffected by capnia, whereas the frequency governing the propagation of RVT variations to neurofluids variations is capnia-dependent. The frequency differences for CSF ROIs can be found in Supplementary Figure S4.

### Interaction between the fMRI and HRV/RVT time series

**FIGURE 9:**
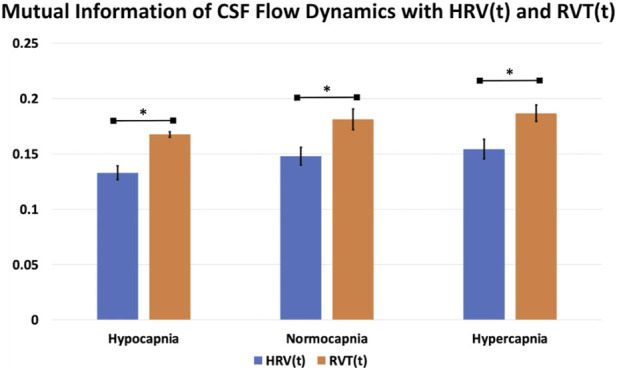
Comparison of mutual information of CSF flow dynamics with *HRV(t)* and *RVT(t)*. Blue: *HRV(t)*; orange: *RVT(t)*. The error bars represent the standard error across participants and the asterisk indicates significant differences.

To further understand the contribution of respiratory and cardiac variability to neurofluid dynamics, we calculated mutual information measures relating HRV and RVT waveforms to neurofluid waveforms. Compared to *HRV*(*t*), *RVT*(*t*) showed higher (p_Hypocapnia_ = 0.03, p_Normocapnia_<0.001, p_Hypercapnia_ = 0.02; D_Hypocapnia_ = 1.1, D_Normocapnia_ = 2.0, D_Hypercapnia_ = 1.1) amounts of mutual information with CSF flow (Figure 9). Nevertheless, the mutual information analysis did not reveal significant differences across capnic conditions. There was, however, a trend of increasing mutual information from hypocapnia to hypercapnia. Details for all ROIs can be found in Supplementary Figure S2.

## Discussion

It is increasingly established that fMRI-based CSF fluctuation measurements reflect variations in hemodynamic. Dynamic properties of these hemodynamic variations can in turn be modulated biomechanically by cerebrovascular reactivity (CVR), neuronally by the ANS or by the effect of CO_2_ on neuronal excitability. However, it remains unclear to what extent each pathway contributes when all three are altered. In this work, we dissected these different mechanisms by measuring neurofluid dynamics as reflected by BOLD during altered steady-state CO_2_, i.e. during hypercapnia, normocapnia and hypocapnic baselines. We demonstrated that the ANS is the primary driver of CSF flow under variations of the steady-state CO_2_, mainly through frequency rather than amplitude. Our main findings are:

1. While CO_2_ can modulate CVR and fluctuations in hemodynamic, the manner of this biomechanical modulation remains unaltered across capnic conditions, and do not drive the variations in neurofluid dynamics across capnias;
2. CO_2_-induced suppression of neuronal activity was not the main driver of differences in neurofluid dynamics across capnias;
3. In addition to respiration, as previously reported, HRV also independently drives low-frequency neurofluid flow as an indication of the ANS pathway of control.
4. Altered CO_2_ alters neurofluid dynamics primarily through the frequency (instead of the amplitude) of heart-rate and respiratory-volume variability.

In this work, we prioritize the derivative-based CSF flow velocity time course for representing CSF dynamics, as it is distinct from the signals from the CSF ROIs, which are in turn more similar to those of arterial and venous ROIs (Figures 5, 6). This is in agreement with our previous findings (Attarpour et al., 2021) as well as with based on cine phase contrast (ElSankari et al., 2013) and highlights the differences in the contrast mechanisms between CSF flow and CSF-fMRI signal (Figure 2).

### The ANS prospective

#### Via respiration and heart rate variability

There are complex interactions between the ANS and neurofluid flow (Halani et al., 2015; Picchioni et al., 2022), as well as between basal CO_2_ and ANS tone. Respiratory variability is a key metric associated with ANS function (Lane et al., 2009; Lehrer and Gevirtz, 2014; Napadow et al., 2008). Increased CO_2_ or decreased O_2_ levels trigger the sympathetic nervous system (SNS) via the chemoreceptors in the brainstem and carotid bodies, resulting in an increase RR and HR to maintain proper gas exchange (Davis et al., 1977), thereby modulating RVT and leading to reduced HRV. Hypercapnia is associated with a more slowly varying RRF (Figure 5), which is reminiscent of the slowly-varying BOLD response that Cohen et al. observed during a hypercapnic baseline (Cohen et al., 2002). In contrast to RVT, HRV mainly modulates neurofluid oscillation through an amplitude modulation, in which hypocapnia results in a higher first peak and a lower second peak (Figure 6). For instance, the flip from positive (in hypocapnia) to negative (in normocapnia) for the first peak (Figure 6) indicates that the direction of flow associations with HRV is also different across capnias. These findings are consistent with changes in RR, HR, RVT and HRV potentially altering both signal fluctuation amplitude and frequency of their corresponding fMRI signal components (Birn et al., 2006; Chang et al., 2009). In fact, in our analysis, RR was found to significantly correlate with rs-fMRI signal fluctuation frequency in neurofluid ROIs in the low-frequency bands (Band 1 and Band IS1) as well as in the GGMS (Band IS1). HR and HRV were also found to be significantly associated with the frequencies of the venous ROI (Band IS2), the GGMS (Band 1–2) and CSF flow (Band IS2). Thus, in this study, we found ANS associations primarily through frequency coupling. A better understanding of the frequency coupling adds a new dimension to previous studies that mainly focused on the temporal pattern of CSF dynamics (Picchioni et al., 2022; Yang et al., 2024).

Despite contributions from both RVT and HRV, our mutual information analysis indicates that RVT exhibits higher mutual information with CSF flow than does HRV, indicating the respiratory system is the main determinant of ANS-CSF modulation. Thus, our results show that the manner in which both HRV and RVT modulate neurofluid dynamics varies by capnia. A broader implication could be that if different individuals can be found on different points on the capnic spectrum, basal capnia could also be an important contributor to inter-participant and inter-session variability in RRF, as well as in neurofluid flow patterns.

#### Via vascular tone

**FIGURE 10:**
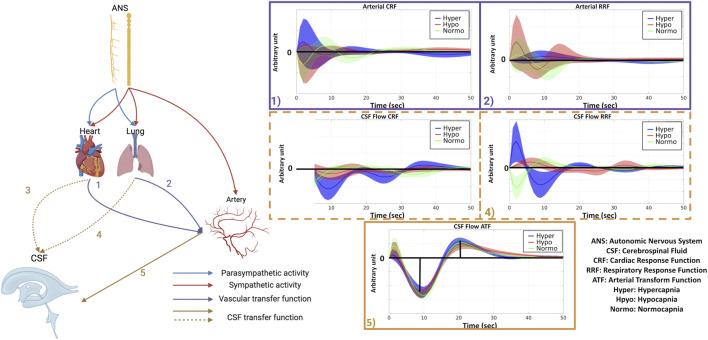
An overview of the pathways that drive the flow of CSF by the ANS. The lung and heart both receive parasympathetic (blue arrow) and sympathetic (red arrow) input, whereas the arterial vasculature potentially only receives sympathetic input (red arrow). The lung and the heart may have a direct impact on arterial BOLD (purple arrows 1 and 2), which is modulated by arterial CRF (1) and RRF (2). The CSF flow is directly driven by vascular pulsation (brown arrow 5), modulated by CSF flow ATF (5), as well as indirectly by heart (blue dashed arrow 3) and lung (blue dashed arrow 4) activity, which are modulated by CSF flow CRF (3) and RRF (4). (Figure partially based on biorender: https://www.biorender.com/). Hyper (blue): hypercapnia; hypo (red): hypocapnia; normo (green): normocapnia.

Similar to the heart and lung, the microvasculature also receives input from the ANS, more specifically the sympathetic nervous system (Figure 10). In fact, previous findings by Peebles et al. supported the regulation of CVR by the sympathetic nervous system through the alpha1-adrenoreceptors (Peebles et al., 2012). Elevated SNS tone triggers the release of norepinephrine from sympathetic nerve endings near blood vessels. Norepinephrine binds to alpha-adrenergic receptors on smooth muscle cells in the blood vessel walls, causing them to contract. This vasoconstriction also leads to increased mean arterial pressure (MAP) (Smith and Maani, 2023) and reduced cerebral blood flow (CBF), increasing vascular tone (Jordan et al., 2000). In the brain, this vasoconstriction may also divert blood towards the heart and limbs, except for in regions related to the stress response (ter Laan et al., 2013). A recent study suggests a negative correlation between SNS activity and peripheral vasomotion in anticipation of pain stimuli (Xu et al., 2024). Thus, the proposed vascular-tone driven pathway of ANS influence can be summarized as: increased CO_2_ → increased SNS tone → reduced CBF and hemodynamic → reduced vasomotion. However, this is not what we observed, as we observed higher neurofluid oscillation amplitude at higher CO_2_ levels. Thus, the interplay between the ANS and neurofluid dynamics does not appear to be dominated by ANS control of vascular tone.

### ANS-independent respiration and cardiac pulsation prospective

Aside from the ANS-mediated modulation of CSF dynamics, baseline CO_2_ may modulate CSF dynamics through other potential pathways. Baseline CO_2_ may alter the heart and respiratory rate directly through stimulating chemoreceptors in the brainstem, independently of the ANS (Kronenberg and Drage, 1973). At a group level, while not significant, HR is higher at both hyper- and hypocapnia, while RR is highest at hypercapnia and lowest at hypocapnia. While the group comparisons did not support significance of these differences, HR and RR still differed across conditions on an individual level (Supplementary Figure S1). Such shifts in HR and RR can result in seemingly counterintuitive observations in terms of fMRI frequency. For example, a shift of the HR-related fMRI signal peak to a higher frequency can result in the aliasing of the HR peak into Band 1. However, based on our HR measurements, this potential for aliasing only applies to 3 of the participants (Supplementary Figure S1). Accordingly, based on the limited group effects observed in our experiment, baseline CO_2_ modulation of neurofluid via HR and RR does not appear comparable to the effect of ANS-mediated modulation.

### The biomechanical perspective

While CO_2_ is widely used to alter vascular tone and hemodynamics (Ainslie and Duffin, 2009; Cohen et al., 2002), which are in turn tightly linked to CSF dynamics, the mechanisms through which basal CO_2_ influences CSF dynamics are less well documented. Of particular relevance to the present study, CO_2_ can modulate vascular tone, and hence hemodynamic fluctuations, independently of the ANS. However, it has been unclear how much basal CO_2_ influences CSF dynamics solely through its biomechanical effect on CVR.

Vascular tone is conventionally defined as the baseline level of constriction in a blood vessel relative to its maximally dilated state. However, the ability of fMRI signals to fluctuate depends not only on dilatory capacity but also on constrictive capacity. Thus, in the context of BOLD based neurofluid fluctuation measurements, a more neutral vascular tone (i.e. during normocapnia) is biomechanically associated with the highest level of signal fluctuations, as was shown in our previous work (Halani et al., 2015). This is consistent with previous work by Biswal et al., who uncovered a suppression of 0.08 Hz band rs-fMRI signal fluctuations during 5% hypercapnia, which was attributed to the reduced sensitivity of hemodynamic modulation during a vasodilated state (Biswal et al., 1997). These observations can in part explain our own observations of significant positive correlations between rs-fMRI signal fluctuation amplitude and CVR, with CVR being diminished at hyper- and hypocapnia compared to normocapnia (Golestani et al., 2016). Moreover, previous work by Cohen et al. (2002) found that the BOLD peak height was inversely correlated with basal CO_2_, while the BOLD response width was directly correlated with basal CO_2_, implicating a fMRI frequency modulation by CO_2_ as well.

As hemodynamic fluctuation amplitude reflects CVR, the biomechanical hypothesis would predict that the higher CVR at normocapnia be associated with higher CSF fluctuations than at hyper- or hypocapnia, and that the hypocapnic baseline be associated with faster CSF fluctuations (as shown in Figure 1). The results of our study, however, were very different. Instead, we found CSF as well as vascular fluctuation amplitude and frequency to both be higher at hyper- and hypocapnia (Figure 8). Our findings are also in accordance with previous findings in which it was found that ANS rather than (or in addition to) local CO_2_ plays a crucial role in modulating cerebral arterial blood flow (Özbay et al., 2019). Moreover, given the ATF is unchanged across capnias (Figure 7), implying that the ability of arterial pulsations to drive CSF pulsations is independent of vascular tone and CVR, the biomechanical mechanism is unlikely to dominate the differences in the dynamics of CSF across capnias.

### The neuronal activity prospective

An alternate or complementary mechanism for the effect of CO_2_ on the rs-fMRI signal is through the effect of CO_2_ on neural activity. Elevated CO_2_ has been associated with suppressed steady-state amplitude of the band-limited EEG Hilbert envelope amplitude in multiple EEG and MEG bands (Driver et al., 2016; Xu et al., 2011). By the same token, reduced CO_2_ should be associated with enhanced EEG amplitude. In this regard, previous work suggests that neuronal activity variations can influence hemodynamics, which in turn influence CSF fluctuations as measured using BOLD rs-fMRI. Fultz et al. (2019) found that the global grey-matter BOLD signal was coupled with the CSF fMRI signal from the fourth ventricle, which was in turn coupled with low-frequency delta activity. Thus, if hypercapnia and hypocapnia act to suppress and enhance synchronized neuronal activity fluctuations, respectively, they can also enhance and suppress the CSF signal fluctuations, respectively.

In this experiment, contrary to this expectation, we did not see a reduction in CSF fluctuations at higher CO_2_ levels (also illustrated in Figure 1, and shown in (as seen in Supplementary Figure S3; Supplementary Table S1)). Although we also investigated the capnia-dependence of the GGMS, which has been associated with global neuronal activity (Schölvinck et al., 2010; Huber et al., 2024), the GGMS has also been linked with systemic physiological, hemodynamic fluctuations (Power et al., 2017; Tong et al., 2013) as well as ANS activity (Özbay et al., 2019), particularly within the low-frequency range (Band 1).

### Implication of CSF fluctuation amplitude and frequency

Current literature has been focused on the amplitude (i.e., power) of neurofluid fluctuations (Yang et al., 2024; Picchioni et al., 2022), so it remains unclear what the frequency of these fluctuations mean. As mentioned earlier, due to the vasogenic drivers, the infraslow bands (IS1 and IS2) are most strongly associated with ANS control (Figure 4). What is the role of slow CSF flow fluctuations? While the fast, cardiac-driven pulsations are considered the primary engine for the convective bulk flow that powers the glymphatic system, the slower oscillations in CSF, such as those related to ANS modulation, might have a modulatory effect on this convective flow (Kedarasetti et al., 2022). Moreover, slow-wave flow can maximize the exchange between the CSF and interstitial fluid that enables waste removal.

A recurring finding in this work is the influence of capnic condition on neurofluid fluctuation frequency in this slow band, as shown in Figure 8. In this low-frequency range, modulating CO_2_ levels appears to modulate the frequency response of the respiratory control of neurofluid fluctuations. Since our RR was maintained across all capnic conditions (Table 1), and since RR itself does not necessarily correlate with low-frequency variations in RR (which is measured by RVT), a more interesting question is “what underlies the link between frequencies of *HRV*(*t*), *RVT*(*t*) and neurofluid signal frequency?”, which remains to be clarified in future research.

### Limitations

One of the major limitations of this study may be the limited number of participants. However, although our dataset provides unique insight into the physiological activity during different capnic conditions, only 13 participants were included, which limits the generalizability of the results from linear mixed effect models. In addition, participants were excluded for the aqueduct (n = 1) and fourth ventricle (n = 4) ROIs, which necessitates more cautious interpretation of results from these two regions. Further, we used a relatively low spatial resolution in order to maximize the temporal sampling rate. While we believe that a high sampling rate is essential for an analysis of this nature, we realize the potential for partial-volume effects in our analysis. Additionally, due to the limited field of view, ROIS for the aqueduct and fourth ventricle analysis may not be available in all participants (also see *Methods* section). Finally, despite our efforts to maximize the sampling rate, our temporal resolution of 380 ms may not fully capture all cardiac harmonics.

In our study, we chose macrovascular and CSF ROIs that are distant from sites of local neural activity. In spite of this, the impact of the capnic condition on global neural activity cannot be entirely ignored (Xu et al., 2011). There is a possibility that variations in global neural activity can be transmitted into macrovasculature, which supplies and drains brain blood. In our linear-mixed effect model, these factors have not been included. In addition, several other factors, such as vasomotion and vascular tone, are difficult to monitor directly and have not been incorporated into the linear mixed effect model. It is also noteworthy that the macrovascular ROIs used in this study are based on regions with high fMRI-signal power, which may differ from real macrovascular structures based on recent biophysical models (Zhong et al., 2024).

Peripheral physiological recordings provide insight into cardiac and respiratory activity, and we used them as a surrogate for the activity of the autonomic nervous system. Numerous studies have demonstrated the link between ANS activity and cardiac and respiratory activity; however, the modulation of cardiorespiratory processes depends on far more than ANS activity alone (Gordan et al., 2015). Yet, as we included only healthy young participants and used short capnic periods, the ANS may be the most likely pathway for cardiac and respiratory variation in the present study. Nevertheless, the interplay of different physiological systems on neurofluids dynamics across different capnias should not be overlooked and should be investigated in future studies.

## Conclusion

The study of neurofluid dynamics using *in-vivo* imaging is an exciting new avenue of research that provides important insights into waste clearance and neuronal signalling in the brain. In this work, we investigate the relationship between capnic conditions and neurofluid dynamics as measured by BOLD signal fluctuations. Inducing different levels of basal CO_2_ has well-established effects on cerebrovasculature, which are in turn linked to the flow of CSF. However, in this study, we found this link is tightly associated with autonomic activity. Moreover, we found that neurofluid dynamics are very similar to those of the global mean signal. We believe these findings can provide new insights into the regulation of neurofluid flow, especially of CSF flow.

## Data Availability

The raw data supporting the conclusions of this article will be made available by the authors, without undue reservation.
