# Supplementary material for: Modulation of neurofluid fluctuation frequency by baseline carbon dioxide in awake humans: the role of the autonomic nervous system
Source: Front Physiol. 2026 Feb 18;17:1750101. doi: 10.3389/fphys.2026.1750101 (PMC12956733; doi:10.3389/fphys.2026.1750101)
Supplement: Supplementary file 1 [file DataSheet1.pdf]

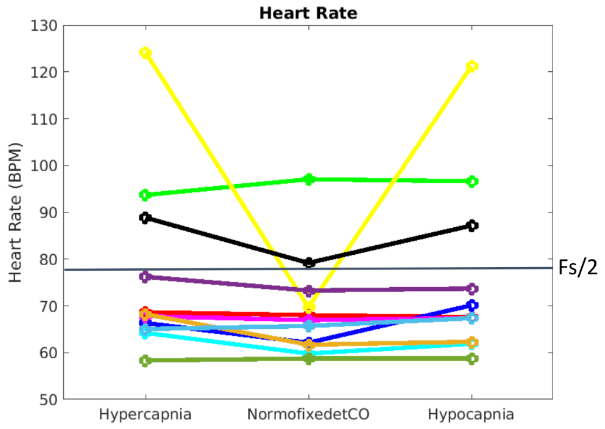

Figure S1. The participant-level trends across all capnic conditions for HR (left) and RR (Right). Different colours represent different participants.

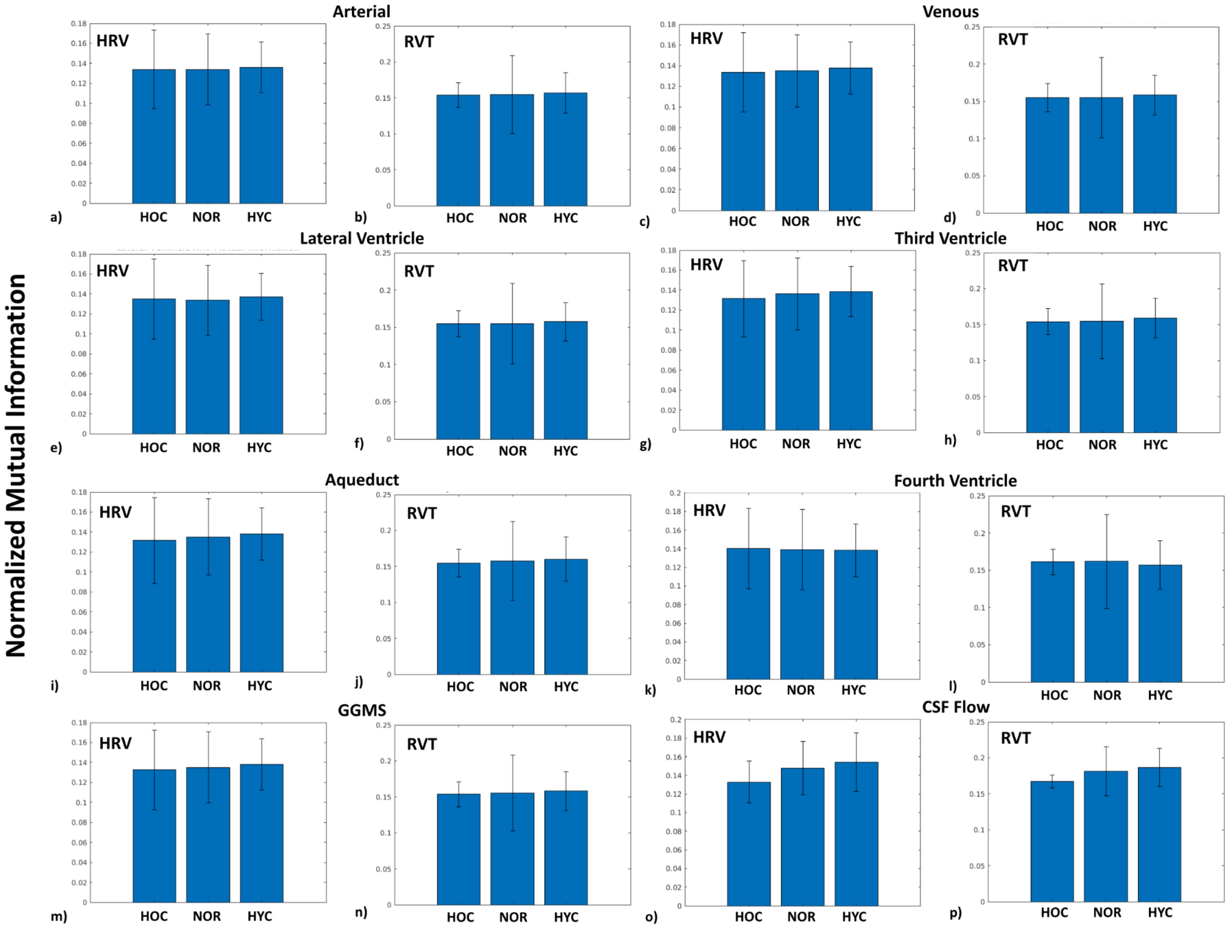

Figure S2. Mutual Information between HRV, RVT and the ROI-specific BOLD signals across all capnic conditions. The error bars represent inter-participant standard deviations.

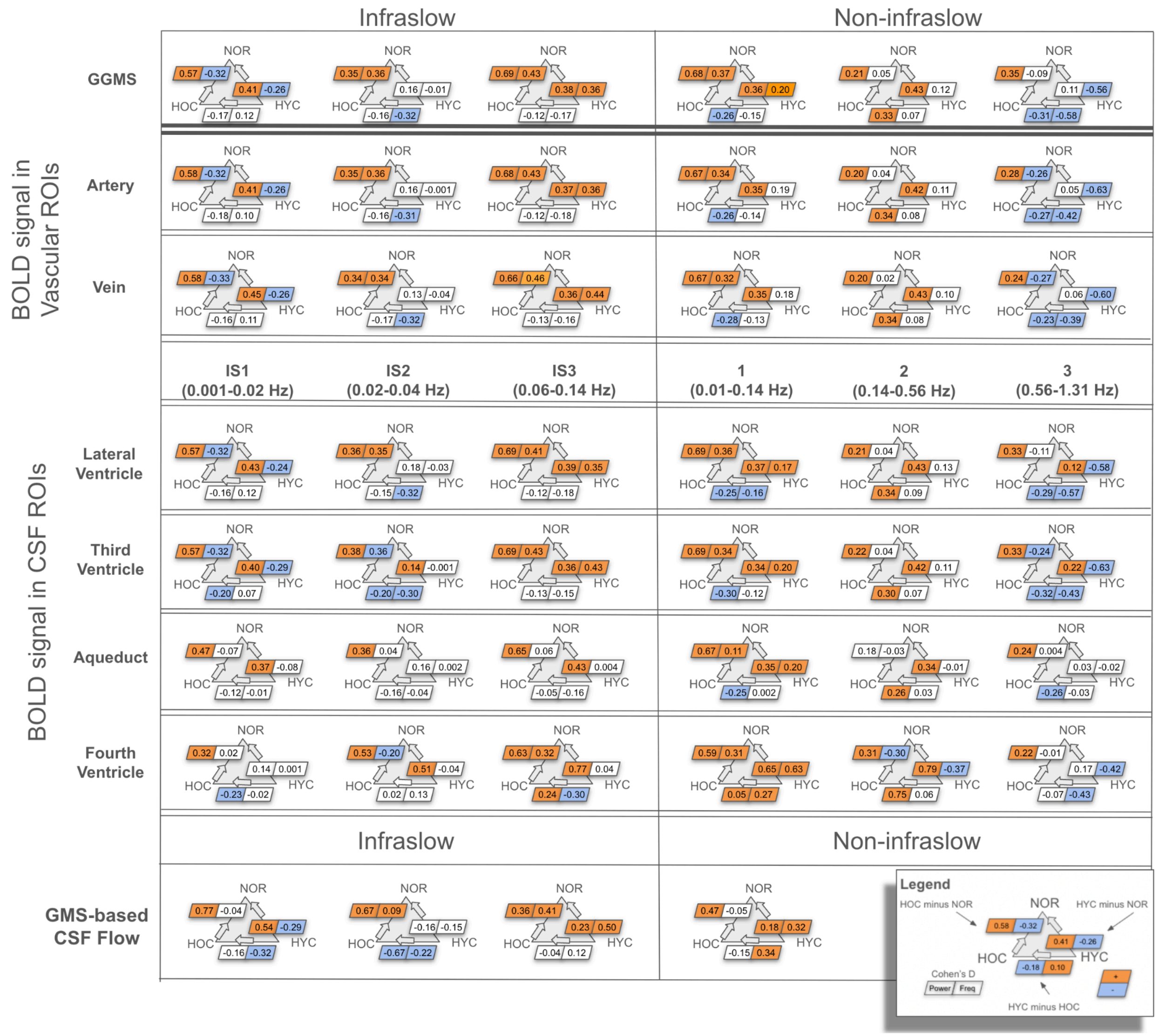

Figure S3. Effect sizes of differences in rs-fMRI oscillation power and frequency across different capnias. NOR: normocapnia; HOC: hypocapnia; HYC: hypercapnia. The figure depicts three infraslow bands (IS1, IS2 and IS3) and three non-infraslow bands (1, 2 and 3) for vascular ROIs (artery and vein), CSF ROIs (lateral ventricle, third ventricle, aqueduct and fourth ventricle) and GMS-based CSF flow. We have thresholded the D values in order to present only effects greater than 'small'.

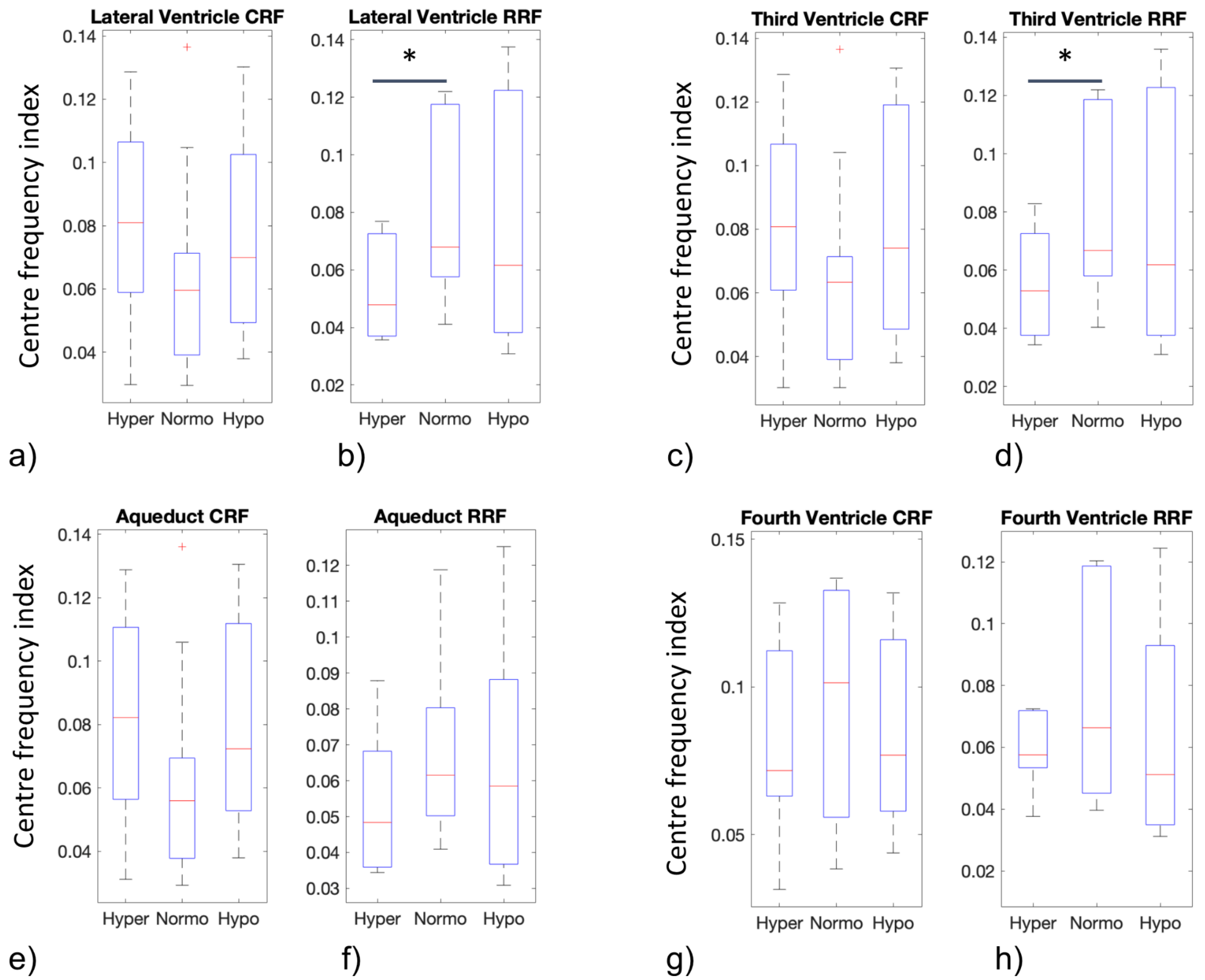

Figure S4. Summarize the centre-frequency indices of RRF and CRF calculated for the rs-fMRI signals in the CSF ROIs (lateral ventricle a-b, third ventricle c-d, aqueduct e-f and fourth ventricle g-h). Asterisks indicate significant differences based on the Wilcoxon signed test ($p < 0.05$). Hyper: hypercapnia; Hypo: hypocapnia: Normo: normocapnia.

Table S1. An overview of rs-fMRI oscillations across different capnias. Results from neurofluid ROIs and CSF flow are summarized in reference to those from the grey-matter-specific GMS (GGMS). The results were color-coded according to whether BOLD signal power is higher (pink), lower (powder blue), and whether BOLD signal frequency is higher (orange) or lower (blue) in each comparison.

	Hypocapnia, as compared to normocapnia (HOC minus NOR)	Hypercapnia, as compared to normocapnia (HYC minus NOR)	Hypercapnia, as compared to hypocapnia (HYC minus HOC)
GGMS BOLD	Higher BOLD signal power in all frequency bands<br>Lower BOLD signal frequency in Band IS1 and 3<br>Higher BOLD signal frequency in Band IS2, IS3 and 1	Higher BOLD signal power in all frequency bands except for Band 3<br>Lower BOLD signal frequency in Band IS1 and 3<br>Higher BOLD signal frequency in Band IS3	Higher BOLD signal power in Band 2<br>Lower BOLD signal power in Band IS2, 1 and 3<br>Lower BOLD signal frequency in Band IS2 and 3<br>Higher BOLD signal frequency in Band 2 (artey only)
BOLD in vascular ROIs	Higher BOLD signal power in all frequency bands<br>Lower BOLD signal frequency in Band IS1 and 3<br>Higher BOLD signal frequency in Band IS2, IS3 and 1	Higher BOLD signal power in all frequency bands except for Band 3<br>Lower BOLD signal frequency in Band IS1 and 3<br>Higher BOLD signal frequency in Band IS3	Higher BOLD signal power in Band 2<br>Lower BOLD signal power in Band IS2, 1 and 3<br>Lower BOLD signal frequency in Band IS2 and 3<br>Higher BOLD signal frequency in Band 2 (artey only)
BOLD in CSF ROIs	Higher BOLD signal power in all frequency bands<br>Lower BOLD signal frequency in Band IS1 and IS2 (3rd and 4th ventricle)<br>Higher BOLD signal frequency in Band IS2 (lateral ventricle), IS3 and 1	Higher BOLD signal power in all frequency bands (except for Band 3 in the aqueduct and 4th ventricle)<br>Lower BOLD signal frequency in Band IS1 and 3<br>Higher BOLD signal frequency in Band IS3 and 1 (except for aqueduct and 4th ventricle)	Higher BOLD signal power in Band 2<br>Lower BOLD signal power in all frequency bands except for Band IS3 and 2<br>Lower BOLD signal frequency in Band 1 and 3<br>Higher BOLD signal frequency in Band IS2 (3rd ventricle only)

**GMS -based CSF Flow**	**Higher CSF-velocity power in all frequency bands**<br>**Higher CSF-velocity fluctuation frequency in Band IS2 and IS3**	**Higher CSF-velocity fluctuation power in all frequency bands except for Band IS2**<br>**Lower CSF-velocity fluctuation frequency in Band IS1**<br>**Higher CSF-velocity fluctuation frequency in Band IS2 and 1**	**Lower BOLD signal power in Band IS2**<br>**Higher CSF-velocity fluctuation frequency in all bands except for Band IS3**
